# Thin-Slice Measurement of Wisdom

**DOI:** 10.3389/fpsyg.2017.01378

**Published:** 2017-08-15

**Authors:** Chao S. Hu, Michel Ferrari, Qiandong Wang, Earl Woodruff

**Affiliations:** ^1^Institutes of Psychological Sciences, Hangzhou Normal University Hangzhou, China; ^2^Centre for Cognition and Brain Disorders, Hangzhou Normal University Hangzhou, China; ^3^Zhejiang Key Laboratory for Research in Assessment of Cognitive Impairments Hangzhou, China; ^4^Applied Psychology and Human Development Department, University of Toronto, Toronto ON, Canada; ^5^Peking-Tsinghua Center for Life Sciences, Peking University Beijing, China

**Keywords:** Thin-slice, measurement of wisdom, Thin-slice wisdom Paradigm, Berlin Wisdom Paradigm, Universal Wisdom Paradigm

## Abstract

Objective Measurement of Wisdom within a short period of time is vital for both the public interest (e.g., understanding a presidential election) and research (e.g., testing factors that facilitate wisdom development). A measurement of emotion associated with wisdom would be especially informative; therefore, a novel Thin-Slice measurement of wisdom was developed based on the Berlin Paradigm. For about 2 min, participants imagined the lens of a camera as the eyes of their friend/teacher whom they advised about a life dilemma. Verbal response and facial expression were both recorded by a camera: verbal responses were then rated on both the Berlin Wisdom criteria and newly developed Chinese wisdom criteria; facial expressions were analyzed by the software iMotion FACET module. Results showed acceptable inter-rater and inter-item reliability for this novel paradigm. Moreover, both wisdom ratings were not significantly correlated with Social desirability, and the Berlin wisdom rating was significantly negatively correlated with Neuroticism; feeling of surprise was significantly positively correlated with both wisdom criteria ratings. Our results provide the first evidence of this Thin-slice Wisdom Paradigm’s reliability, its immunity to social desirability, and its validity for assessing candidates’ wisdom within a short timeframe. Although still awaiting further development, this novel Paradigm contributes to an emerging Universal Wisdom Paradigm applicable across cultures.

## Introduction

Political leaders (e.g., Lincoln, Mao Zedong) are among the most frequently nominated wisdom exemplars in both Western and Eastern cultures although both political systems and wisdom criteria vary across different cultures (Hu et al., 2016; Weststrate et al., 2016). However, these lay people’s nomination may be influenced by irrational factors, e.g., political leaders’ facial attractiveness (Zebrowitz et al., 2015). Therefore, a vital issue for democracy is to measure wisdom objectively, especially within a short period of time. In fact, rapid objective measurement of personal wisdom is also important for wisdom experts who hope to promote wisdom development. However, to date, wisdom research has been limited by methods that rely on self-report (e.g., Ardelt’s three-dimensional wisdom scale) and verbal think-aloud procedures (e.g., Berlin Wisdom Paradigm) that allow people to give socially desirable answers.

A method that allows real-time emotion assessment by human raters, or even artificial intelligence, may minimize the chance for social desirability and thus be a more objective measure of wisdom. In fact, emotion plays an important role in the development and utilization of wisdom (Sternberg and Jordan, 2005; Ardelt and Ferrari, 2014). A recent review reported that emotional homeostasis was commonly cited as a component of wisdom (Bangen et al., 2013). However, very few empirical studies have been conducted on the emotional aspect of wisdom.

Kunzmann and Baltes (2003) measured participants’ self-report of affective feelings and found negative correlations of wisdom score to negative and pleasant feelings in the past year and thus proposed studying the relationship between wisdom and actual emotional reactions to specific emotion-arousing events. Recently, Thomas and Kunzmann (2013) extended the Berlin scenario to real-life video clips of marital conflicts; however, they relied on the participants’ verbal report to measure emotional reactions, which is equally vulnerable to social desirability, especially when asked to describe or explain their thoughts and behavior, rather than simply verbalizing their silent thoughts about the problem (Ericsson and Simon, 1993).

The traditional Berlin Wisdom Paradigm assessed participants’ responses to hypothetical life-scenario situations usually irrelevant to their personal life and thus less emotionally engaging than real life situations (Baltes and Staudinger, 2000). For example, “In reflecting over their lives, people sometimes realize that they have not achieved what they had once planned to achieve. What should one/they do and consider?” (Staudinger and Baltes, 1996, p. 762). Participants reflected on these questions for a while before they responded by thinking aloud. Their responses were recorded, transcribed, and finally rated by 10 well-trained raters on five Berlin wisdom criteria: Declarative knowledge, procedural knowledge, value relativism, lifespan contextualism and management of uncertainty (Staudinger et al., 1994).

Ardelt (2004) critiqued the Berlin Wisdom Paradigm’s definition of wisdom as expert knowledge, arguing that wisdom cannot exist independently of individual people. Based on Clayton and Birren’s definition of wisdom, Ardelt (2003, 2004) proposed that wisdom involves the integration of cognitive, reflective, and affective personality qualities. Ardelt developed a 39 items self-report/self-assessment scale to measure wisdom on these three dimensions, not unlike many assessments of personality traits (e.g., Eysenck Personality Traits Scale [1975]). For each item, participants indicate on a 5-point response scale from “Definitely true of myself” or “strongly agree” to “Definitely not true of myself” or “strongly disagree” (Ardelt, 2003, 2004).

Although these two Western wisdom paradigms are well-known and have been used internationally, studies have shown that cultures differ in how wisdom is understood (e.g., Ferrari et al., 2011; Brezina and Oudenhoven, 2012; Hu et al., 2016). In general, Eastern understanding of wisdom emphasizes both analysis and synthesis, whereas Western understandings of wisdom generally emphasize analysis (Takahashi and Bordia, 2000). Western researchers studying wisdom are arguably influenced by Western culture and have mainly studied Western participants growing up in a Western cultural background (for reviews, see: Sternberg and Jordan, 2005; Bangen et al., 2013; Glück et al., 2013); therefore, their scientific models of wisdom may not be relevant for other cultural groups.

Bang and Zhou (2014) argued that prototypical Chinese wisdom was generally congruent with that in Ardelt’s wisdom model, and conducted a survey among Chinese young adults using Ardelt’s three-dimensional wisdom scale. However, factor analysis failed to identify the three dimensional model proposed by Ardelt, and instead found a 4-dimensional structure involving “non-dualistic thinking,” “perspective-taking,” “non-resentment,” and “empathy.” In fact, our previous survey with 97 Chinese adults (46 males, aged from 18 to 23 years, *M* = 19.71, *SD* = 1.19 years) also failed to identify the three dimensional model proposed by Ardelt, the model in the 12-Item Abbreviated Three-Dimensional Wisdom Scale (Thomas et al., 2015), or the four-factor structure proposed by Bang and Zhou (2014) (For the details of our survey, see the Supplementary Tables.).

Moreover, the Lie/Social desirability score (measured within Eysenck personality scale) was significantly positively correlated with Ardelt wisdom score (*r* = 0.26, *p* = 0.037), confirming Ericsson and Simon’s (1993) and Staudinger and Glück’s (2011) proposition that self-reported measurement of psychological constructs may be influenced by self-serving bias and inaccurate self-perception. In fact, a positive correlation between impression management and scores on some items in the Ardelt (2003) three-dimensional wisdom scale was already demonstrated in a previous study (Taylor et al., 2011).

Considering above theoretical rationales and empirical findings, we conducted this study to develop a measurement of wisdom that would allow measurement of real-time emotional reactions and be suitable for cross-culture studies. A performance-based measurement was developed by adapting the Berlin Wisdom Paradigm.

The traditional Berlin Wisdom Paradigm relies on an objective third-person perspective when measuring wisdom performance, using vignettes potentially irrelevant to participants’ personal lives. Mickler and Staudinger (2008) extended the original Berlin paradigm and argued that ‘general wisdom’ as measured in the traditional Berlin Wisdom Paradigm differed from Personal Wisdom, considered an individual’s insight into his or her own life. However, again following Ericsson and Simon (1993), there is good reason to believe that their first-person perspective wisdom performance measure may be as vulnerable to social desirability and inaccuracy in self-judgment as is Ardelt’s measure. For this reason, we adopted a position between these two extremes and developed a second-person perspective wisdom performance measure, in which participants imagined the lens of a camera as the eyes of their friend/teacher whom they advised about pre-designated life dilemmas framed in the Berlin wisdom vignettes.

Our 2nd person task is not meant to replicate the Berlin paradigm tasks but to adapt them to this new approach. The original Berlin paradigm used vague problems to elicit participant’s meta-level thinking rather than their concrete advice, which is one of the key aspect critics like Ardelt (2004) have leveled against it. Our aim is to strike a balance between general 3rd person considerations provoked by the original Berlin paradigm and 1st person approaches that are more emotionally engaging but also liable to social desirability bias. Ericsson and Simon (1993) underlined that research participants were simply verbalizing the information they attend to while generating an answer to a problem (which is what happens in the 2nd person method) instead of describing, explaining, justifying, or rationalizing their actions (which is what happens in the 1st and 3rd person methods), and thus the 2nd person method may be less vulnerable to social desirability bias.

We expected that talking to an imagined friend when left alone in a quiet room should be less affected by social desirability, compared with thinking-aloud about oneself being a friend. Although the task of speaking to a camera as if another person may seem unnatural, and so not ecologically valid, it is actually modeled on the ‘empty chair’ technique developed in the Gestalt therapy that has been empirically proven to be of therapeutic value despite its artificiality, because it evokes a much deeper and authentic response to life problems (Paivio and Greenberg, 1995; Wagner-Moore, 2004) —and therefore, we suggest, one probably less subject to social desirability—than simply describing or explaining what must be done (as required in the original Berlin paradigm) or oneself as a friend (as in Staudinger’s personal wisdom paradigm). It is for this reason, too, that we can expect the emotions generated and captured by the camera to be an important non-verbal indicator meaningfully associated with wisdom. In fact, many Thin-slice studies have demonstrated the accuracies in ratings of personality and intelligence from videotaped episodes that lasted only for a few minutes or even seconds; an accuracy in intelligence rating not influenced by stereotypes of gender and age (Ambady and Rosenthal, 1993; Borkenau et al., 2004).

Furthermore, the traditional Berlin wisdom vignettes are implicitly based on Western cultural contexts that may not be suitable for measuring wisdom among the Chinese. A previous study extending the Berlin paradigm has already demonstrated that wise reasoning about fundamental life issues is related to the relevance of problems in individuals’ own lives (Thomas and Kunzmann, 2013). Thus, wisdom vignettes based on Chinese social and cultural life might be more ecologically valid than the original Berlin vignettes for Chinese participants. For these reasons, two culturally appropriate scenarios were used in our paradigm: “a friend’s unrealizable dream,” a relatively common life scenario for contemporary Chinese adults; and “a teacher’s unrealized dream,” adapted from a classic Berlin scenario.

Since the Berlin wisdom Criteria also may not be perfectly applicable to Chinese participants, a Chinese Wisdom Criteria (see **Table 1** for details) was developed based on our previous study exploring Chinese implicit theory of wisdom (Hu et al., 2016): 50 older Chinese (age 60–84 years) and 50 younger (age 20–30 years) participants first nominated personal acquaintances and historical figures as wisdom exemplars and then gave their own definition of wisdom from which five latent factors were identified. In order to compare the Berlin criteria and our Chinese criteria, our participants’ responses were rated on both sets of criteria by 20 well-trained raters.

**Table 1:** Chinese wisdom criteria and their definitions.

Wisdom component	Definition	Sample quotations
Cognitive engagement	Motivation to engage in cognition and reflection about the external world and the internal mind; skill in such cognition, and the outcome of these cognitive processes.	“They (singers) may not be as happy as they appear to be”. “There is no standard criterion to measure whether you realize your dream or not”. “You have educated us diligently and contributed much, and yet you say ‘life is meaningless,’ which I can sympathize with: your once busy life has suddenly become idle”.
Practical engagement	Motivation and ability to successfully put into practice the outcome of cognitive engagement.	“You could teach singing to the elders in your neighborhood”. “You could participate in some competitions for singers, and improve your singing”. “You need more practice. Even if your skill in singing would improve in the future, it would take a long time”.
Social engagement	Motivation and ability to engage with others for the goodness of everyone.	“Your song brings happiness to us, your friends”. “You need to consider your family”. “You need to consider others’ potential negative opinion if you still want to become a singer”. “You could tell us students about your dream, and we will realize it for you”.
Spirituality of disengagement	Disengage oneself from civilization and worldly issues and return to the primitive nature or “Buddha”: the ultimate truth.	“If you are only seeking fame, social status and wealth (through becoming a singer), I would advise you to quit” “If you realize your dream, you may suddenly feel life is meaningless”.
Positive mindset	In face of the hardship in life, exert oneself to overcome the difficulty and improve oneself, through satisfaction with life.	“Competition in today’s society is so fierce that if your mental strength is firm enough I would encourage you to fearlessly strive for your dream”. “If you keep seeking your dream, you may feel your life is meaningful, even if it is never realized”.

In addition, facial expressions during each wisdom response were videotaped and then analyzed for emotional reactions. The effect of emotion on physical expression is faster, more automatic, less controlled and thus less contaminated by social desirability than that on verbal speech. In fact, Paul Ekman and Friesen (2003) argue that authentic emotions behind facial expressions can be detected through combined analyses of multiple facial muscular actions of transient subtle micro-expressions. Following Kunzmann and Baltes (2003), we hypothesized that emotional reactions (i.e., positive and negative emotion) of individuals with wisdom—understood within the Berlin Paradigm as expertise in the fundamental pragmatics of life—should be more prevalent than that of individuals without this life expertise.

Finally, in order to test whether the participants’ tendency toward social desirability would affect their wisdom performances, in light of general personality traits, they completed the Eysenck Personality scale before taking our wisdom measurement.

### Hypotheses

The wisdom vignettes based on Chinese social and cultural life should be more reliable than the Berlin vignette adapted for Chinese participants when assessing wisdom performance in China. Nevertheless, the wisdom rating on the Chinese wisdom criteria should be positively correlated with the Berlin Wisdom criteria, due to similarities between the Berlin wisdom model and Chinese implicit theory of wisdom that reflect broad cultural universals about wisdom. Positive and negative emotional reactions during the wisdom performance should be more prevalent in responses rated as wiser. Finally, social desirability score should not be significantly correlated with either the Berlin or the Chinese wisdom rating.

## Materials and Methods

All procedures used in the current study were approved by the Ethic Committees at Zhejiang Normal University and the University of Toronto. Sample size was selected to allow for quantitative analyses with Type I error rate set at 0.05. Written consent was not obtained from our participants, because they had given their recorded verbal consent to participate, and given their names and contact information before finishing our survey; moreover, we had explained the nature of our study, assured them they could stop at any time without penalty, and ensured that their personal information would be kept private. This consent procedure was approved by the Ethic Committees.

### Participants

Thirty undergraduate students (12 males) at Zhejiang Normal University aged from 18 to 21 (*M* = 19.10, *SD* = 0.75) participated in this study. All subjects were native Chinese speakers and had normal or corrected-to-normal vision. Each participant was compensated 20 RMB (about 3 United States dollars).

### Material

**The Chinese version of shortened Eysenck personality scale (Qian et al., 2000)** measures Psychoticism, Extroversion, Neuroticism/emotional instability, and Lie/Social desirability scales: Psychoticism is the tendency to develop psychotic symptoms or anti-social behavior; Extraversion is the tendency to be active in social activities; Neuroticism measures individuals’ emotional instability and the tendency to develop negative feelings; the Lie scale contains questions about which individuals tend to lie due to social desirability. Reliability tests revealed that Cronbach’s alpha for these subscales was mixed: Neuroticism: 0.75; Extroversion: 0.71; Lie/Social desirability: 0.59; and Psychoticism: 0.21.

#### Berlin Style Wisdom Vignettes

Two fundamental life scenarios were printed on an A4 paper: (1) The “life review problem” adapted from a Berlin scenario: *“Imagine one of your teachers suddenly feels life is meaningless because he has not realized a dream he always had since his youth. What would you say to him?”*; and (2) the “Life plan problem” was invented by the first author as potentially more common in real lives of contemporary Chinese students, while remaining true to the Berlin Wisdom Paradigm action-theoretical approach, in which wisdom is involved in life planning, management and review: *“Imagine one of your friends dreams of becoming a singer, but he is really bad at singing and is not young anymore; if he asked you how he sings, what would you say to him?”*

Answers were scored analogously to the ***Berlin Manual instructions (Staudinger et al., 1994)***, ignoring several aspects of the original Berlin manual instructions due to the particulars of our research setting; in particular, the think-aloud procedure was not used, given our 2nd-person approach to measuring wisdom, and thus the sections related to the think-aloud procedure training were replaced with an analogous 2nd-person practice task. Furthermore, ***Chinese wisdom criteria*** were developed based on a previous study on implicit theory of wisdom among Chinese (Hu et al., 2016).

In addition, we used a***laptop with a camera*** that captured 29 frames per second of emotional reactions, which were later analyzed in the ***iMotions – Attention Tool FACET module (version 2.1)***. Previous studies have confirmed a link between the facial affect data of the FACET module to established peripheral arousal measures such as event-related potentials (ERP), heart rate variability (HRV), and galvanic skin response (GSR) (Amico et al., 2016).

### Procedure

Participants finished the Eysenck personality scale about one or 2 months before taking our Thin-slice wisdom measurement.

The participants completed the two vignette scenario assessments in a quiet laboratory room individually. They were instructed to read the vignettes printed on an A4 paper while sitting in an armchair, with the laptop camera set about 30 cm away from their face. The participants were asked to imagine the camera as the eyes of their friend/teacher and talked to “him/her” while their responses were videotaped by the camera. Before the formal task, participants underwent a practice task (i.e., talking to a friend who had become bankrupt) to become accustomed to the unusual situation of talking to a camera. In order to mimic a natural situation, there was no set time limit for participant responses. During both the reflection and answer time, the experimenter left the laboratory room so that the participants could feel less constrained in their performance.

### Data Analysis

#### Wisdom Ratings

Twenty graduate students in the Department of Psychology at Zhejiang Normal University capable of reading academic papers in psychology written in Chinese and English were recruited and trained according to the Berlin Wisdom Manual. Each rater was compensated 200 RMB (about $30 United States). Ten raters (7 females, ages 23–27, Mean age = 24, *SD* = 1.33) were randomly chosen to rate the videotaped responses on the Berlin Wisdom criteria; another 10 raters (6 females, ages 24–28, Mean age = 26, *SD* = 1.20) rated the videotaped responses on the Chinese wisdom criteria. To prevent mutual influence from the ratings on different criteria (“halo effect”), each rater rated videos on only one criterion; in order to calculate the inter-rater reliability, two raters rated each criterion.

Our raters were adequately trained according to the manual (e.g., scale anchoring, rating each transcript independently rather than ranking them, using training videos). All raters engaged in a practice wisdom rating (rating a videotape of the first author’s advice concerning the advantages and disadvantages for a Chinese student to study abroad for a doctorate degree) before rating the participants’ videotaped performance. Each rater rated the videotaped performance in random order. Participants’ responses ranged from 14 to 502 seconds (*M* = 88 s).

#### Facial Expression Analysis

The Attention Tool FACET calculates the probable occurrence of seven basic emotions (joy, sadness, anger, contempt, fear, surprise, and disgust) and three general valences (negative, positive, and neutral). This system first identifies and locates the face in each frame of the video; and then automatically measures basic facial Action Units (AU) listed in the well-established Facial Action Coding System (FACS), a comprehensive, compositional, and anatomically based facial muscular movement analysis system (Ekman and Rosenberg, 1997). Quoting the FACET module Manual, “It is useful to think of facial expressions as words and of AUs as the letters that make up those words. For example, one of the most common expressions of fear contains a combination of AU1 [Brow Raiser Frontalis], AU2 [Outer Brow Raiser Frontalis], AU4 [Brow Lowerer Depressor Glabellae], and AU5 [Upper Lid Raiser Levator Palpebrae Superioris].”

FACET module provided an evidence value for each emotion category on each frame of each video. The aggregate time of the positive evidence frames for a category of emotion per video was used as an ideal estimation of the aggregate time of that emotion, since the chance that a frame was mistaken as emotional equals the chance that an emotional frame failed to be identified.

In order to compare the aggregate time of an emotion across responses of varying duration, we calculated the “proportional time of emotion” by dividing the number of positive evidence frames by the total number of frames within a video, excluding ineffectively analyzed frames. For example, the response of one female participant for the first Berlin-Style Wisdom vignette lasted for about 88 s and generated 2565 effectively analyzed frames of which 27 were positive on surprise; thus, the proportional time of surprise was 27 divided by 2565, or about 1.05% percent.

## Results

### Reliability of the Berlin and Chinese Wisdom Ratings

In the life plan problem, “a friend’s unrealizable dream,” the reliability between the two raters was analyzed for each criterion and inter-rater reliability was acceptable for all the Berlin criteria (Cronbach’s Alpha > 0.60). Overall Cronbach’s alpha for the Berlin paradigm was computed by treating the 10 individual ratings as items, Cronbach’s Alpha > 0.70. Therefore, these ratings were averaged to get the final Berlin Wisdom rating, ranging from 1.80 to 5.10 (*M* = 3.19, *SD* = 0.91).

Inter-rater reliability was acceptable for all the Chinese criteria, except for the Chinese wisdom criterion “Spirituality of disengagement,” which was probably difficult for the student raters to comprehend; two middle-aged raters were then recruited to rate the participants’ performances in the life plan problem on “Spirituality of disengagement”: One was a 35-year-old male associate professor in Psychology at Zhejiang Normal University; the other was a 33-year-old male editor for Zhejiang University Publisher. They were not financially rewarded for their ratings, but were promised a research report of this study. Inter-rater reliability for this new rating was acceptable (Cronbach’s alpha = 0.83), therefore, the average rating from these middle-aged raters was adopted as the final rating on “Spirituality of disengagement.” Overall Cronbach’s alpha for the Chinese criteria ratings was computed by treating the 10 individual ratings as items, Cronbach’s Alpha > 0.70. These ratings were then averaged to get the final Chinese Wisdom rating, ranging from 2.90 to 6.00 (*M* = 4.37, *SD* = 0.67).

In the life plan problem “a friend’s unrealizable dream,” the Berlin Wisdom rating was significantly positively correlated with the Chinese Wisdom rating, *r* = 0.73, *p* < 0.001. Pearson correlations among and between the ratings on each of the Berlin and Chinese wisdom criteria in the life plan problem are shown in **Table 2**.

**Table 2:** Correlations for ratings on the Berlin and Chinese wisdom criteria.

Wisdom criteria	Practical engagement	Positive mindset	Social engagement	Spirituality of disengagement	Factual knowledge	Procedural knowledge	Contextualism	Value relativism	Uncertainty
Cognitive engagement	0.51^∗∗^	–0.17	0.73^∗∗^	0.50^∗∗^	0.66^∗∗^	0.74^∗∗^	0.77^∗∗^	0.68^∗∗^	0.76^∗∗^
Practical engagement		0.54^∗∗^	0.46^∗^	0.18	0.42^∗^	0.43^∗^	0.19	0.22	0.50^∗∗^
Positive mindset			–0.17	–0.35	–0.09	–0.34	–0.47^∗∗^	–0.40^∗^	–0.12
Social engagement				0.44^∗^	0.86^∗∗^	0.84^∗∗^	0.71^∗∗^	0.58^∗∗^	0.67^∗∗^
Spirituality of disengagement					0.41^∗^	0.50^∗∗^	0.64^∗∗^	0.40^∗^	0.43^∗^
Factual knowledge						0.78^∗∗^	0.73^∗∗^	0.54^∗∗^	0.58^∗∗^
Procedural knowledge							0.79^∗∗^	0.67^∗∗^	0.72^∗∗^
Contextualism								0.74^∗∗^	0.69^∗∗^
Value relativism									0.58^∗∗^

*^∗^*p* < 0.05, ^∗∗^*p* < 0.01.*

Because “Positive Mindset” was negatively correlated with the ratings on other wisdom criteria overall, this wisdom component was removed from our model, and the Chinese wisdom rating was calculated by averaging the ratings on the other four Chinese wisdom criteria, ranging from 3.13 to 6.25 (*M* = 4.43, *SD* = 0.79). This newly calculated Chinese wisdom rating was significantly positively correlated with the Berlin wisdom rating, *r* = 0.87, *p* < 0.001. For the following analyses, this Chinese wisdom rating based on the model of cognitive, practical, social engagements and spirituality of disengagement was adopted.

In the life review problem (“a teacher’s unrealized dream”), inter-rater reliability was not acceptable for most of the Berlin and Chinese criteria; therefore, no wisdom rating was calculated for this problem.

### Relationship between Emotion Reactivity and Wisdom Performance

Twenty-nine participants’ effectively measured frames were more than 84% of the total frames in the videos (*M* = 99.11%, *SD* = 2.58%). [The facial expression of one participant was not effectively measured (54.59%), probably because she did not look directly at the camera]. Therefore, data of these 29 participants were included in the following analyses. The proportional time of each category of emotion in each scenario was listed in **Table 3**. Paired sample *t*-tests revealed no significant difference in any category of emotion between the performances in life plan and life review problems.

**Table 3:** Proportional time of emotion during the participants’ performance in different scenarios (unit: percent).

	A friend’s unrealizable dream				A teacher’s unrealized dream			
Emotion	Minimum	Maximum	Mean	*SD*	Minimum	Maximum	Mean	*SD*
								
Joy	0.0	91.4	9.5	18.1	0.0	34.4	6.1	4.5
Anger	0.0	93.7	17.0	24.5	0.0	100.0	14.3	28.0
Surprise	0.0	65.7	5.9	12.8	0.0	66.3	4.1	13.3
Fear	0.0	60.4	15.8	21.3	0.0	75.7	15.9	20.7
Contempt	0.0	74.1	10.4	18.7	0.0	78.7	10.0	16.6
Disgust	0.0	100.0	33.3	36.1	0.0	100.0	30.4	35.1
Sadness	0.0	61.0	12.3	19.1	0.0	80.5	14.1	22.8
Neutral	0.0	100.0	48.6	28.6	0.0	100.0	50.4	29.7
Positive	0.0	93.8	21.1	25.5	0.0	53.3	16.6	15.1
Negative	0.8	100.0	70.8	30.7	0.8	99.9	72.2	30.4

In order to test whether participants’ emotion reactivity was related to their wisdom performance, Spearman correlation analyses^1^ were conducted between the proportional time of each emotion (seven basic emotion categories and three valences) and the Berlin and Chinese Wisdom ratings. Results showed that the proportional time of surprise was significantly positively correlated with both the Berlin and Chinese Wisdom ratings (See the Supplementary Figures for the scatterplots); other correlations were not significant (see **Table 4**).

**Table 4:** Spearman correlations of Chinese and Berlin wisdom ratings with different emotions during the scenario “A friend’s unrealizable dream.”

	Chinese	Berlin
Joy	0.12	0.00
Anger	–0.18	–0.04
Surprise	0.55ˆ**	0.50ˆ**
Fear	0.23	0.33
Contempt	0.05	0.06
Disgust	–0.05	–0.08
Sadness	0.14	0.09
Neutral	–0.06	–0.08
Positive	0.16	0.02
Negative	–0.21	–0.09

*^∗^*p <* 0.05, ^∗∗^*p <* 0.01.*

#### Relationship between Emotion Reactivity and Response Length

Response length was considered a meaningful outcome of the wisdom performance in a previous study (Mickler and Staudinger, 2008), therefore, Spearman correlation analyses were conducted to examine the relationship between emotion reactivity and response length. Proportional time of surprise was significantly positively correlated with response length in both life dilemma tests: “a friend’s unrealizable dream” (*rho* = 0.41, *p* = 0.029) and “a teacher’s unrealized dream” (*rho* = 0.46, *p* = 0.013).

### Correlations with Social Desirability

Pearson correlation analysis revealed that neither the proportional time of emotion, nor the wisdom ratings were significantly correlated with Social desirability score, all *p* > 0.05. Nevertheless, the Berlin Wisdom rating was significantly negatively correlated with “Neuroticism,” *r* = -0.47, *p* = 0.024 (*N* = 23).

## Discussion

Our results showed acceptable inter-rater and inter-item reliabilities for our Thin-Slice Wisdom Paradigm. Moreover, this novel approach’s minimization of social reliability was partially confirmed by the null correlation between the wisdom ratings and Social desirability score in the Eysenck Personality scale. Of course, participants still knew that they were talking to a camera and that their responses might be evaluated, so their responses might still differ from what they would actually say in a real-life situation. Nevertheless, our novel paradigm provides a more ecological approach capturing more emotional aspects, compared with other methods.

### The Roles of Wisdom Criteria and Wisdom Vignette in Performance Measurement

The Berlin wisdom rating was highly consistent with the Chinese Wisdom rating. In fact, previous studies have demonstrated that wisdom rating by raters’ own understanding of a wise response (Global Wisdom Rating) is highly consistent with ratings on the Berlin criteria (Staudinger et al., 1992; Zacher et al., 2015). Taken together, these results suggested that performance measures of wisdom are relatively robust across different rating systems. On the other hand, the “life review problem” vignette adapted from the Berlin wisdom vignette was not reliable, perhaps because advising a retiring teacher is an unusual life scenario for Chinese undergraduate students. Therefore, designing appropriate vignettes with which participants have experience was arguably much more important than selecting culturally specific wisdom criteria for wisdom measurement across different cultures.

Although only one Chinese wisdom criterion “Cognitive engagement” is related to the Berlin wisdom criteria, the Chinese wisdom ratings were mostly significantly positively correlated with the Berlin wisdom ratings (see **Table 2**), probably because wisdom is a perfect integration of different psychological components—as demonstrated by the significant positive correlations among different Chinese wisdom components—and thus the level of one wisdom component (e.g., cognitive engagement) can predict the levels of other wisdom components (e.g., practical engagement, social engagement).

### The Role of Experience in Wisdom Rating

In the life review problem, “a teacher’s unrealized dream,” inter-rater reliability was unacceptable on almost every wisdom criterion, perhaps because these undergraduate students had no experience of advising a teacher on any life problem, something potentially considered improper in Chinese culture. Likewise, although inter-rater reliability was unacceptable between the student raters on the Chinese wisdom criterion “Spirituality of disengagement,” it was acceptable between middle-aged academic raters; this suggests that raters’ life experience is important for accurate rating on an elusive criteria about a profound life philosophy, one that may require a certain amount of life experience to understand. In fact, previous researchers have suggested that even well-trained student raters were not as good as middle-aged academics raters, with inter-rater correlations among student raters being lower than those among middle-aged academic raters (Glück et al., 2013).

“Spirituality of disengagement” may be implicated in the implicit theory of wisdom among the middle-aged and older raters but not student raters, making comprehension of “Spirituality of disengagement” more difficult for them. Still, the ratings of these middle-aged raters were moderately positively correlated with one student rater, respectively, *r* = 0.44, *r* = 0.55, but not the other, all *r* < 0.15. Therefore, some student raters may have a more developed implicit theory of wisdom, that allows them to comprehend this wisdom criterion just as do the middle-aged academic raters.

### Emotion, Wisdom, and Aging

The significantly negative correlation between neuroticism and Berlin wisdom rating was consistent with the common theory that emotional homeostasis is a subcomponent of wisdom (Bangen et al., 2013). Moreover, with this Thin-slice Wisdom Measurement we found a significant positive correlation between surprise and wisdom when discussing a hypothetical scenario. Although our sample was not large, our effect size was fairly large: both *rho* > 0.50 (between the wisdom ratings and proportional time of surprise). With a larger sample size, it would have been more likely to detect a significant correlation.

Feeling of surprise may be the beginning of wisdom. Individuals usually do not question or ponder their original thoughts until they feel surprised by unexpected information: either from external environment or from their internal reaction to their own thoughts. Since feeling surprised is positively correlated with feeling of difficulty—something considered important for metacognition (Touroutoglou and Efklides, 2010), itself believed to be important for wisdom (Sternberg, 2001)—surprise may incite individuals to wonder about and then to reflect upon their original thoughts, thereby arriving at a deeper understanding of what is being discussed. Perhaps a greater feeling of surprise provokes greater cognitive effort, as demonstrated by the significant correlation between response length and feeling of surprise in our results.

By contrast, “Positive Mindset” was generally negatively correlated with wisdom ratings on other criteria (see **Table 2**), especially with the Berlin Criteria of “Contextualism” and “Value Relativism” (*r* = -0.47, *r* = -0.40, all *ps* < 0.05); thus, “Positive Mindset” may not be a valid component of Chinese wisdom. Wiser individuals are probably more willing to process negative information, even if this undermines their positive feelings. By contrast, less wise individuals are more willing to process and recall the positive information, a “positivity bias” that might increase when getting older, evidenced by previous studies on emotion recognition and memory (Baddeley et al., 2015; Di Domenico et al., 2015; Altamura et al., 2016). Wiser individuals may have less “positivity bias” as they age, and thus show less positive emotion when addressing difficult life problems.

### Relevance for Cross-Cultural Studies of Wisdom

In general, our studies provided intriguing results for wisdom researchers who plan to conduct cross-cultural studies of wisdom, suggesting the need to consider cultural differences in both the conception and structure of wisdom. As revealed in our studies, the Berlin wisdom vignette may not be applicable to participants from non-Western cultures. Nevertheless, we should expect some universal aspects of wisdom to be shared by all human beings, since some fundamental life problems are shared by people in every nation (e.g., the life plan problem of an “unrealizable dream”).

An ambitious but meaningful project may be to identify common life problems through a review of autobiographies across different cultures, perhaps of wisdom exemplars. We hypothesize that similarities in fundamental life problems across cultures should predominate, despite some cultural differences. Moreover, even though people in different cultures may respond differently to these fundamental life problems, we expect some latent universal criteria to be evident in their performance; a wise response to such universal life problem may be considered wise across different cultures and explain the timelessness and near-universal appeal of some historical wisdom exemplars like Buddha.

In cross-cultural wisdom research projects, performance-based wisdom measurement is probably a more appropriate method for effectively identifying universal and culturally specific wisdom components and criteria. Videotaped wisdom performance of participants can be rated on different systems of wisdom criteria, based on different wisdom models. For example, wisdom criteria could be developed from Ardelt’s three-dimensional wisdom model and participants’ wisdom performance in different nations rated on Ardelt’s wisdom dimensions, just as they were on the Berlin wisdom criteria and our Chinese wisdom criteria. Eventually, researchers could use factor analysis to identify the common factors and combine these wisdom criteria into universal criteria, thereby contributing to the development of a Universal Wisdom Model and Paradigm.

Perhaps the most important innovation in our study was the Thin-slice wisdom measurement: rating videotaped wisdom performances in which participants performed as if they were really addressing some fundamental life problem by talking to someone personally familiar to them. Although such a performance was fictional, as in Gestalt therapy, performers’ habitual thinking, emotion, and action should reveal a habitual Gestalt and expressed through their emotional reactions.

Non-verbal wisdom components are necessarily missing in transcript responses commonly used to assess wisdom performance, therefore, examining a person’s emotional expression associated with their speech seems a more authentic way to assess wisdom performance. In fact, insight and homeostasis conveyed in the facial expressions of wisdom exemplars’ (e.g., Buddha) advice-giving may be integral to the expression of wisdom across cultures.

### Limitations

Our participants were all Chinese undergraduate students; the Ardelt’s scale might work better assessing older Chinese adults. Also, facial expression analysis technology is still under development, just as any computer technology. In addition, our Chinese Wisdom Paradigm is not without its own limitations. For example, our Thin-slice Wisdom Measurement is still not an entirely ecological measure: The participants were talking to a camera, which was unnatural for them. Furthermore, participants might offer different advice to different listeners. Likewise, a larger set of scenarios involving different life problems would provide a more comprehensive and reliable measurement of wisdom. Finally, although relatively small, a sample size of 30 is a typical size for this methodology given the enormous amount of data generated by each subject and each of the 20 raters. A more economical paradigm would be needed for a larger sample.

## Conclusion

In general, our results provided the first evidence for the Thin-slice Wisdom Paradigm’s reliability, minimization of social desirability, and validity for quickly assessing candidates’ wisdom. A more developed Thin-slice Wisdom Measurement could serve both the public interest (e.g., understanding a presidential election) and research needs (e.g., testing factors facilitating wisdom development). Although the Thin-slice Wisdom Paradigm is admittedly still rudimentary and awaiting further development, we hope our fledgling attempt to develop this novel measurement paradigm will contribute to wisdom studies in different cultures through its innovative approach to assessing wisdom performance.

## Author Contributions

CH, MF, and EW contributed to the conceptualization of this study. CH designed the study, analyzed the data, and wrote this research report. QW conducted the study and collected the data.

## Conflict of Interest Statement

The authors declare that the research was conducted in the absence of any commercial or financial relationships that could be construed as a potential conflict of interest.

## Footnotes

1 The distribution of an emotion’s proportional time is not normal, thus Pearson correlation analyses were not suitable.
