# Supplementary material for: Thin-Slice Measurement of Wisdom
Source: Front Psychol. 2017 Aug 15;8:1378. doi: 10.3389/fpsyg.2017.01378 (PMC5559494; doi:10.3389/fpsyg.2017.01378)
Supplement: Supplementary file 1 [file Table_1.docx]

Supplementary Material

Adaptation of Western Wisdom Paradigms and development of a 2nd person measurement for Chinese

Chao S. Hu *, Michel Ferrari, Qiandong Wang, Earl Woodruff

*** Correspondence:** Chao S. Hu: chao.super.hu@gmail.com

# Supplementary Tables

**1.1 Ardelt Wisdom Scale used in Study 1.**

| **No.** | **Chinese Item** | **Original Item** | **Dimension** |
| --- | --- | --- | --- |
| 1 | 在我们生活的复杂世界里，我们了解发生了什么事情的唯一渠道就是依赖那些可以信赖的领袖或专家。 | In this complicated world of ours the only way we can know what’s going on is to rely on leaders or experts who can be trusted | COGNITIVE |
| 2 | 我讨厌那些只知自责、埋怨自己而生活不开心的人。 | I am annoyed by unhappy people who just feel sorry for themselves | AFFECTIVE |
| 3 | 生活在大多数时候基本都一样。 | Life is basically the same most of the time | COGNITIVE |
| 4 | 人们太过于关注动物的感受。 | People make too much of the feelings and sensitivity of animals | AFFECTIVE |
| 5 | 几乎所有的人都能够被分为诚实和欺诈两类。 | You can classify almost all people as either honest or crooked | COGNIVITE |
| 6 | 如果我目前的境况有所改变我将会感觉好很多。 | I would feel much better if my present circumstances changed | REFLECTIVE |
| 7 | 做任何事情的正确方法都只有一种。 | There is only one right way to do anything | COGNITIVE |
| 8 | 我知道有些人我永远都不会喜欢。 | There are some people I know I would never like | AFFECTIVE |
| 9 | 对于那些无法改变的事情，知道的太多反而不好。 | It is better not to know too much about things that cannot be changed | COGNITIVE |
| 10 | 事情经常在我没有做错任何事的情况下还会出错。 | Things often go wrong for me by no fault of my own | REFLECTIVE |
| 11 | 无知是福。 | Ignorance is bliss | COGNITIVE |
| 12 | 与各种各样的人在一起我都感觉舒服。 | I can be comfortable with all kinds of people | AFFECTIVE(reversed) |
| 13 | 一个人要么知道问题的答案要么不知道。 | A person either knows the answer to a question or he/she doesn’t | COGNITIVE |
| 14 | 如果其他人有麻烦而且需要帮助，与我无关。 | It’s not really my problem if others are in trouble and need help | AFFECTIVE |
| 15 | 人要么是好人要么是坏人。 | People are either good or bad | COGNITIVE |
| 16 | 我试图考虑争论各方的立场之后才做决定。 | I try to look at everybody’s side of a disagreement before I make a decision | REFLECTIVE(REVERSED) |
| 17 | 如果看到需要帮助的人，我总会以某种方式努力去帮助他。 | If I see people in need, I try to help them one way or another | AFFECTIVE(reversed) |
| 18 | 当我因某人烦恼时，我通常试着花些时间从他的角度看问题。 | When I’m upset at someone, I usually try to “put myself in his or her shoes” for a while | REFLECTIVE(REVERSED) |
| 19 | 有些人我特别不喜欢，当他们因为自己做过的事被人逮住、受到惩罚时，我心里就很高兴。 | There are certain people whom I dislike so much that I am inwardly pleased when they are caught and punished for something they have done | AFFECTIVE |
| 20 | 我总是尽量考虑问题的方方面面。 | I always try to look at all sides of a problem | REFLECTIVE(REVERSED) |
| 21 | 有时候我真地同情每一个人。 | Sometimes I feel a real compassion for everyone | AFFECTIVE(REVERSED) |
| 22 | 我试图预料并且避免那些有可能使我不得不努力思考的情境。 | I try to anticipate and avoid situations where there is a likely chance I will have to think in depth about something | COGNITIVE |
| 23 | 当我回顾发生在自己身上的事情时，总忍不住感到愤恨。 | When I look back on what has happened to me, I can’t help feeling resentful | REFLECTIVE |
| 24 | 当别人需要被安慰时，我经常不去安慰。 | I often have not comforted another when he or she needed it | AFFECTIVE |
| 25 | 如果我认为某个问题没有解决办法，那它对我来说没有多少吸引力。 | A problem has little attraction for me if I don’t think it has a solution | COGNITIVE |
| 26 | 如果事情出错了，我要么特别生气要么特别沮丧。 | I either get very angry or depressed if things go wrong | REFLECTIVE |
| 27 | 我有时不会因为别人有困难而太难过。 | Sometimes I don’t feel very sorry for other people when they are having problems | AFFECTIVE |
| 28 | 我常常不理解人们的行为。 | I often do not understand people’s behavior | COGNITIVE |
| 29 | 有时我会被情感左右，以至不能想到多少解决问题的办法。 | Sometimes I get so charged up emotionally that I am unable to consider many ways of dealing with my problems | REFLECTIVE |
| 30 | 有时就在别人和我讲话时，我会希望他们离开。 | Sometimes when people are talking to me, I find myself wishing that they would leave | AFFECTIVE |
| 31 | 我更喜欢让事情顺其自然地发生，而不是试图弄明白它们为什么会那样。 | I prefer just to let things happen rather than try to understand why they turned out that way | COGNITIVE |
| 32 | 当我因为某个问题迷惑时，我首先做的一件事就是调查情况、考虑所有相关的信息。 | When I am confused by a problem, one of the first things I do is survey the situation and consider all the relevant pieces of information | REFLECTIVE(REVERSED) |
| 33 | 我不喜欢听别人说自己的烦恼。 | I don’t like to get involved in listening to another person’s troubles | AFFECTIVE |
| 34 | 思考过后，我还会在重大决策上犹豫不决。 | I am hesitant about making important decisions after thinking about them | COGNITIVE |
| 35 | 在责备他人之前，我会试着想象如果自己在他的处境上会有什么感觉。 | Before criticizing somebody, I try to imagine how I would feel if I were in their place | REFLECTIVE(REVERSED) |
| 36 | 我很容易被那些跟我争论的人惹怒。 | I’m easily irritated by people who argue with me | AFFECTIVE |
| 37 | 当我回顾那些发生在我身上的事情时，我会觉得自己被骗了。 | When I look back on what’s happened to me, I feel cheated | REFLECTIVE |
| 38 | 只知道问题的答案，而不知道为什么是这个答案，对我来说没什么。 | Simply knowing the answer rather than understanding the reasons for the answer to a problem is fine with me | COGNITIVE |
| 39 | 我发现有时候从别人的角度看问题很难。 | I sometimes find it difficult to see things from another person’s point of view | REFLECTIVE |

**1.2 Ardelt Wisdom Scale item means and response distributions**

| Item Number | Minimum | Maximum | Mean | Std. Deviation |
| --- | --- | --- | --- | --- |
| 1 | 1 | 5 | 1.98 | 0.79 |
| 2 | 1 | 5 | 3.72 | 1.13 |
| 3 | 1 | 5 | 3.16 | 1.11 |
| 4 | 1 | 5 | 2.18 | 0.82 |
| 5 | 1 | 5 | 2.17 | 0.87 |
| 6 | 1 | 5 | 3.36 | 0.95 |
| 7 | 1 | 2 | 1.52 | 0.50 |
| 8 | 1 | 5 | 3.34 | 1.22 |
| 9 | 1 | 5 | 3.15 | 1.09 |
| 10 | 2 | 5 | 3.34 | 0.85 |
| 11 | 1 | 5 | 2.26 | 1.01 |
| 12 | 1 | 5 | 2.40 | 0.81 |
| 13 | 1 | 5 | 2.69 | 0.99 |
| 14 | 1 | 3 | 1.94 | 0.56 |
| 15 | 1 | 5 | 2.02 | 0.88 |
| 16 | 2 | 5 | 3.75 | 0.79 |
| 17 | 2 | 5 | 3.63 | 0.70 |
| 18 | 2 | 5 | 3.44 | 0.78 |
| 19 | 1 | 5 | 3.11 | 0.87 |
| 20 | 1 | 5 | 3.49 | 0.86 |
| 21 | 1 | 5 | 3.29 | 1.05 |
| 22 | 1 | 5 | 3.26 | 0.89 |
| 23 | 1 | 5 | 2.62 | 0.93 |
| 24 | 1 | 5 | 2.48 | 0.92 |
| 25 | 1 | 5 | 2.79 | 1.00 |
| 26 | 1 | 5 | 2.80 | 1.02 |
| 27 | 1 | 5 | 3.27 | 0.77 |
| 28 | 1 | 5 | 2.93 | 0.93 |
| 29 | 1 | 5 | 3.42 | 0.95 |
| 30 | 1 | 5 | 3.26 | 0.89 |
| 31 | 1 | 5 | 3.12 | 0.92 |
| 32 | 2 | 5 | 3.35 | 0.87 |
| 33 | 1 | 5 | 2.65 | 0.95 |
| 34 | 1 | 5 | 3.74 | 0.89 |
| 35 | 1 | 5 | 3.31 | 0.87 |
| 36 | 1 | 5 | 3.04 | 1.01 |
| 37 | 1 | 4 | 2.74 | 0.85 |
| 38 | 1 | 5 | 2.44 | 1.00 |
| 39 | 1 | 5 | 3.25 | 0.99 |

**1.3 Inter-correlations of all items in the Ardelt Wisdom Scale**

**Left Panel:**

| Item No. | 1 | 2 | 3 | 4 | 5 | 6 | 7 | 8 | 9 | 10 | 11 | 12 | 13 | 14 |
| --- | --- | --- | --- | --- | --- | --- | --- | --- | --- | --- | --- | --- | --- | --- |
| 1 | 1.00 | 0.08 | 0.18 | 0.18 | 0.01 | 0.02 | 0.35 | 0.03 | 0.18 | -0.12 | 0.13 | 0.14 | -0.06 | 0.18 |
| 2 | 0.08 | 1.00 | 0.05 | 0.10 | 0.17 | -0.14 | -0.23 | 0.12 | 0.14 | 0.12 | 0.15 | 0.16 | -0.17 | -0.01 |
| 3 | 0.18 | 0.05 | 1.00 | 0.24 | 0.13 | 0.01 | 0.29 | 0.30 | 0.38 | -0.01 | 0.35 | 0.10 | -0.22 | 0.06 |
| 4 | 0.18 | 0.10 | 0.24 | 1.00 | 0.36 | 0.06 | 0.17 | -0.18 | 0.09 | 0.12 | 0.08 | 0.30 | 0.05 | 0.11 |
| 5 | 0.01 | 0.17 | 0.13 | 0.36 | 1.00 | 0.13 | 0.22 | 0.03 | 0.06 | 0.15 | 0.07 | 0.26 | 0.18 | 0.12 |
| 6 | 0.02 | -0.14 | 0.01 | 0.06 | 0.13 | 1.00 | 0.11 | 0.06 | -0.14 | 0.05 | -0.17 | 0.02 | 0.25 | -0.09 |
| 7 | 0.35 | -0.23 | 0.29 | 0.17 | 0.22 | 0.11 | 1.00 | -0.03 | 0.09 | -0.03 | 0.28 | -0.02 | -0.11 | 0.48 |
| 8 | 0.03 | 0.12 | 0.30 | -0.18 | 0.03 | 0.06 | -0.03 | 1.00 | 0.25 | 0.14 | -0.11 | -0.06 | -0.03 | 0.12 |
| 9 | 0.18 | 0.14 | 0.38 | 0.09 | 0.06 | -0.14 | 0.09 | 0.25 | 1.00 | 0.32 | 0.28 | 0.01 | 0.04 | 0.04 |
| 10 | -0.12 | 0.12 | -0.01 | 0.12 | 0.15 | 0.05 | -0.03 | 0.14 | 0.32 | 1.00 | -0.12 | 0.05 | 0.22 | -0.08 |
| 11 | 0.13 | 0.15 | 0.35 | 0.08 | 0.07 | -0.17 | 0.28 | -0.11 | 0.28 | -0.12 | 1.00 | -0.12 | -0.19 | 0.17 |
| 12 | 0.14 | 0.16 | 0.10 | 0.30 | 0.26 | 0.02 | -0.02 | -0.06 | 0.01 | 0.05 | -0.12 | 1.00 | -0.01 | -0.05 |
| 13 | -0.06 | -0.17 | -0.22 | 0.05 | 0.18 | 0.25 | -0.11 | -0.03 | 0.04 | 0.22 | -0.19 | -0.01 | 1.00 | -0.20 |
| 14 | 0.18 | -0.01 | 0.06 | 0.11 | 0.12 | -0.09 | 0.48 | 0.12 | 0.04 | -0.08 | 0.17 | -0.05 | -0.20 | 1.00 |
| 15 | 0.10 | 0.09 | 0.08 | 0.19 | 0.57 | 0.12 | 0.16 | -0.06 | 0.02 | -0.04 | 0.13 | 0.18 | 0.32 | -0.09 |
| 16 | -0.19 | 0.31 | 0.03 | -0.11 | 0.00 | -0.10 | -0.17 | 0.07 | 0.11 | 0.13 | 0.13 | 0.02 | -0.03 | -0.23 |
| 17 | 0.14 | 0.12 | 0.07 | -0.09 | -0.05 | 0.12 | -0.09 | 0.00 | -0.13 | -0.06 | -0.07 | 0.06 | 0.06 | -0.50 |
| 18 | -0.02 | 0.11 | 0.10 | 0.24 | -0.02 | -0.14 | -0.11 | 0.07 | 0.01 | 0.08 | 0.12 | 0.14 | 0.00 | -0.33 |
| 19 | 0.02 | 0.02 | -0.01 | 0.02 | -0.05 | 0.18 | -0.10 | -0.01 | -0.04 | 0.05 | -0.14 | -0.04 | 0.04 | -0.03 |
| 20 | -0.05 | 0.07 | 0.08 | 0.07 | -0.16 | -0.03 | -0.19 | -0.01 | -0.08 | 0.07 | 0.09 | 0.03 | 0.12 | -0.22 |
| 21 | -0.14 | -0.11 | -0.04 | 0.10 | 0.20 | 0.08 | -0.14 | -0.10 | -0.05 | 0.07 | -0.08 | 0.16 | 0.03 | -0.16 |
| 22 | -0.10 | -0.04 | 0.11 | 0.07 | 0.28 | 0.24 | -0.03 | 0.16 | -0.07 | 0.08 | -0.07 | 0.05 | 0.16 | -0.06 |
| 23 | -0.07 | -0.29 | -0.02 | 0.12 | 0.35 | 0.27 | 0.10 | -0.06 | -0.05 | 0.05 | -0.02 | -0.04 | 0.30 | 0.03 |
| 24 | -0.13 | -0.04 | 0.09 | 0.18 | 0.16 | 0.27 | 0.12 | -0.04 | 0.07 | -0.07 | 0.04 | -0.08 | 0.25 | 0.16 |
| 25 | 0.15 | -0.05 | 0.41 | 0.17 | 0.06 | 0.14 | 0.13 | 0.00 | 0.20 | 0.05 | 0.25 | 0.09 | 0.07 | 0.02 |
| 26 | 0.23 | 0.13 | 0.07 | 0.11 | 0.24 | 0.17 | 0.13 | 0.11 | 0.31 | 0.15 | 0.08 | 0.04 | 0.23 | 0.18 |
| 27 | -0.07 | 0.01 | -0.06 | 0.16 | -0.21 | -0.01 | -0.09 | -0.01 | -0.09 | 0.03 | -0.04 | -0.19 | -0.24 | 0.05 |
| 28 | 0.01 | -0.05 | 0.04 | 0.31 | 0.17 | 0.07 | 0.00 | -0.02 | -0.04 | 0.02 | -0.03 | -0.04 | 0.11 | 0.18 |
| 29 | 0.14 | 0.08 | -0.01 | 0.00 | 0.13 | 0.04 | -0.09 | 0.22 | 0.11 | 0.35 | -0.26 | -0.17 | 0.12 | -0.12 |
| 30 | -0.01 | 0.08 | -0.11 | -0.04 | 0.15 | 0.01 | -0.18 | 0.08 | 0.00 | 0.18 | -0.03 | 0.02 | 0.00 | -0.12 |
| 31 | 0.24 | 0.30 | 0.24 | 0.08 | 0.10 | -0.02 | 0.23 | 0.09 | 0.26 | -0.02 | 0.25 | 0.23 | -0.12 | 0.25 |
| 32 | -0.03 | -0.01 | -0.06 | 0.15 | -0.20 | -0.24 | -0.17 | 0.05 | -0.04 | -0.04 | -0.01 | 0.05 | 0.06 | -0.15 |
| 33 | 0.04 | 0.25 | 0.15 | 0.34 | 0.29 | 0.07 | 0.08 | -0.02 | 0.06 | 0.00 | 0.26 | 0.05 | -0.16 | 0.27 |
| 34 | 0.02 | 0.06 | 0.05 | -0.07 | -0.01 | 0.00 | 0.00 | 0.22 | 0.24 | 0.21 | 0.07 | -0.35 | 0.08 | 0.02 |
| 35 | -0.10 | 0.24 | 0.11 | 0.06 | 0.03 | -0.03 | -0.30 | 0.36 | -0.03 | 0.15 | 0.02 | 0.17 | 0.00 | -0.27 |
| 36 | 0.14 | 0.07 | 0.20 | 0.06 | 0.03 | 0.03 | -0.08 | -0.07 | 0.20 | 0.03 | 0.06 | 0.08 | 0.06 | -0.07 |
| 37 | 0.10 | 0.01 | -0.02 | 0.08 | 0.00 | 0.09 | 0.06 | -0.11 | 0.03 | 0.39 | -0.06 | 0.12 | 0.05 | -0.02 |
| 38 | 0.13 | -0.04 | 0.34 | 0.26 | 0.15 | 0.24 | 0.30 | 0.08 | 0.00 | -0.12 | 0.10 | 0.09 | -0.11 | 0.12 |
| 39 | 0.04 | 0.15 | 0.20 | 0.13 | 0.34 | -0.27 | 0.12 | 0.13 | 0.12 | -0.02 | 0.08 | 0.15 | -0.16 | 0.09 |

**Middle Panel:**

| 15 | 16 | 17 | 18 | 19 | 20 | 21 | 22 | 23 | 24 | 25 | 26 | 27 | 28 | 29 | 30 |
| --- | --- | --- | --- | --- | --- | --- | --- | --- | --- | --- | --- | --- | --- | --- | --- |
| 0.10 | -0.19 | 0.14 | -0.02 | 0.02 | -0.05 | -0.14 | -0.10 | -0.07 | -0.13 | 0.15 | 0.23 | -0.07 | 0.01 | 0.14 | -0.01 |
| 0.09 | 0.31 | 0.12 | 0.11 | 0.02 | 0.07 | -0.11 | -0.04 | -0.29 | -0.04 | -0.05 | 0.13 | 0.01 | -0.05 | 0.08 | 0.08 |
| 0.08 | 0.03 | 0.07 | 0.10 | -0.01 | 0.08 | -0.04 | 0.11 | -0.02 | 0.09 | 0.41 | 0.07 | -0.06 | 0.04 | -0.01 | -0.11 |
| 0.19 | -0.11 | -0.09 | 0.24 | 0.02 | 0.07 | 0.10 | 0.07 | 0.12 | 0.18 | 0.17 | 0.11 | 0.16 | 0.31 | 0.00 | -0.04 |
| 0.57 | 0.00 | -0.05 | -0.02 | -0.05 | -0.16 | 0.20 | 0.28 | 0.35 | 0.16 | 0.06 | 0.24 | -0.21 | 0.17 | 0.13 | 0.15 |
| 0.12 | -0.10 | 0.12 | -0.14 | 0.18 | -0.03 | 0.08 | 0.24 | 0.27 | 0.27 | 0.14 | 0.17 | -0.01 | 0.07 | 0.04 | 0.01 |
| 0.16 | -0.17 | -0.09 | -0.11 | -0.10 | -0.19 | -0.14 | -0.03 | 0.10 | 0.12 | 0.13 | 0.13 | -0.09 | 0.00 | -0.09 | -0.18 |
| -0.06 | 0.07 | 0.00 | 0.07 | -0.01 | -0.01 | -0.10 | 0.16 | -0.06 | -0.04 | 0.00 | 0.11 | -0.01 | -0.02 | 0.22 | 0.08 |
| 0.02 | 0.11 | -0.13 | 0.01 | -0.04 | -0.08 | -0.05 | -0.07 | -0.05 | 0.07 | 0.20 | 0.31 | -0.09 | -0.04 | 0.11 | 0.00 |
| -0.04 | 0.13 | -0.06 | 0.08 | 0.05 | 0.07 | 0.07 | 0.08 | 0.05 | -0.07 | 0.05 | 0.15 | 0.03 | 0.02 | 0.35 | 0.18 |
| 0.13 | 0.13 | -0.07 | 0.12 | -0.14 | 0.09 | -0.08 | -0.07 | -0.02 | 0.04 | 0.25 | 0.08 | -0.04 | -0.03 | -0.26 | -0.03 |
| 0.18 | 0.02 | 0.06 | 0.14 | -0.04 | 0.03 | 0.16 | 0.05 | -0.04 | -0.08 | 0.09 | 0.04 | -0.19 | -0.04 | -0.17 | 0.02 |
| 0.32 | -0.03 | 0.06 | 0.00 | 0.04 | 0.12 | 0.03 | 0.16 | 0.30 | 0.25 | 0.07 | 0.23 | -0.24 | 0.11 | 0.12 | 0.00 |
| -0.09 | -0.23 | -0.50 | -0.33 | -0.03 | -0.22 | -0.16 | -0.06 | 0.03 | 0.16 | 0.02 | 0.18 | 0.05 | 0.18 | -0.12 | -0.12 |
| 1.00 | -0.02 | 0.12 | 0.00 | -0.14 | -0.17 | 0.14 | 0.21 | 0.35 | 0.10 | 0.19 | 0.26 | -0.32 | 0.16 | 0.15 | 0.14 |
| -0.02 | 1.00 | 0.31 | 0.40 | 0.09 | 0.31 | 0.22 | 0.00 | -0.14 | -0.05 | -0.14 | 0.04 | 0.06 | -0.10 | -0.05 | 0.12 |
| 0.12 | 0.31 | 1.00 | 0.43 | -0.01 | 0.32 | 0.07 | 0.21 | 0.05 | -0.13 | -0.11 | -0.17 | -0.03 | -0.08 | 0.08 | 0.16 |
| 0.00 | 0.40 | 0.43 | 1.00 | -0.03 | 0.35 | 0.10 | 0.05 | 0.02 | -0.12 | -0.02 | -0.21 | -0.04 | -0.04 | -0.19 | 0.20 |
| -0.14 | 0.09 | -0.01 | -0.03 | 1.00 | 0.12 | 0.05 | 0.10 | 0.03 | -0.10 | -0.02 | 0.16 | 0.10 | -0.07 | -0.06 | 0.03 |
| -0.17 | 0.31 | 0.32 | 0.35 | 0.12 | 1.00 | 0.14 | 0.01 | 0.05 | -0.05 | -0.13 | 0.00 | 0.09 | 0.04 | -0.01 | 0.12 |
| 0.14 | 0.22 | 0.07 | 0.10 | 0.05 | 0.14 | 1.00 | 0.24 | 0.28 | 0.10 | 0.06 | 0.02 | -0.24 | 0.13 | -0.07 | 0.01 |
| 0.21 | 0.00 | 0.21 | 0.05 | 0.10 | 0.01 | 0.24 | 1.00 | 0.25 | 0.06 | 0.11 | -0.06 | -0.07 | 0.07 | 0.08 | 0.03 |
| 0.35 | -0.14 | 0.05 | 0.02 | 0.03 | 0.05 | 0.28 | 0.25 | 1.00 | 0.24 | 0.05 | 0.10 | -0.28 | 0.17 | 0.11 | 0.20 |
| 0.10 | -0.05 | -0.13 | -0.12 | -0.10 | -0.05 | 0.10 | 0.06 | 0.24 | 1.00 | 0.29 | 0.17 | -0.16 | 0.31 | -0.15 | 0.00 |
| 0.19 | -0.14 | -0.11 | -0.02 | -0.02 | -0.13 | 0.06 | 0.11 | 0.05 | 0.29 | 1.00 | 0.34 | -0.17 | 0.18 | 0.01 | -0.05 |
| 0.26 | 0.04 | -0.17 | -0.21 | 0.16 | 0.00 | 0.02 | -0.06 | 0.10 | 0.17 | 0.34 | 1.00 | -0.09 | 0.23 | 0.30 | 0.09 |
| -0.32 | 0.06 | -0.03 | -0.04 | 0.10 | 0.09 | -0.24 | -0.07 | -0.28 | -0.16 | -0.17 | -0.09 | 1.00 | 0.08 | 0.18 | 0.08 |
| 0.16 | -0.10 | -0.08 | -0.04 | -0.07 | 0.04 | 0.13 | 0.07 | 0.17 | 0.31 | 0.18 | 0.23 | 0.08 | 1.00 | 0.08 | 0.03 |
| 0.15 | -0.05 | 0.08 | -0.19 | -0.06 | -0.01 | -0.07 | 0.08 | 0.11 | -0.15 | 0.01 | 0.30 | 0.18 | 0.08 | 1.00 | 0.28 |
| 0.14 | 0.12 | 0.16 | 0.20 | 0.03 | 0.12 | 0.01 | 0.03 | 0.20 | 0.00 | -0.05 | 0.09 | 0.08 | 0.03 | 0.28 | 1.00 |
| 0.11 | 0.01 | -0.09 | -0.07 | -0.11 | -0.13 | -0.14 | -0.02 | -0.34 | -0.01 | 0.09 | 0.16 | -0.08 | -0.07 | -0.16 | -0.04 |
| -0.15 | 0.14 | 0.27 | 0.45 | -0.01 | 0.49 | 0.01 | -0.03 | 0.05 | -0.06 | -0.25 | -0.29 | -0.02 | -0.14 | -0.10 | 0.11 |
| 0.27 | -0.09 | -0.19 | -0.07 | -0.18 | -0.09 | 0.10 | 0.29 | 0.09 | 0.27 | 0.26 | 0.11 | -0.01 | 0.16 | 0.01 | 0.14 |
| 0.11 | 0.19 | -0.08 | -0.06 | 0.14 | 0.06 | 0.04 | -0.01 | -0.05 | -0.12 | 0.26 | 0.43 | 0.04 | -0.03 | 0.29 | 0.11 |
| 0.14 | 0.21 | 0.35 | 0.33 | -0.14 | 0.27 | 0.08 | 0.03 | -0.01 | -0.06 | 0.01 | -0.02 | -0.08 | 0.09 | 0.06 | 0.28 |
| 0.03 | 0.04 | 0.11 | -0.08 | 0.14 | 0.25 | 0.00 | 0.07 | 0.20 | 0.10 | 0.03 | 0.21 | 0.22 | -0.06 | 0.14 | 0.30 |
| 0.06 | -0.05 | -0.11 | -0.02 | 0.01 | 0.09 | 0.06 | 0.08 | 0.21 | 0.10 | 0.07 | 0.04 | -0.07 | -0.03 | 0.21 | 0.31 |
| 0.10 | -0.05 | -0.12 | -0.13 | -0.04 | -0.19 | -0.05 | 0.06 | -0.05 | 0.24 | 0.39 | 0.19 | 0.02 | 0.30 | 0.02 | 0.02 |
| 0.28 | -0.01 | 0.04 | 0.08 | -0.12 | -0.12 | 0.09 | 0.22 | 0.04 | -0.02 | 0.16 | 0.11 | -0.09 | 0.10 | 0.20 | 0.24 |

**Right Panel:**

| 31 | 32 | 33 | 34 | 35 | 36 | 37 | 38 | 39 |
| --- | --- | --- | --- | --- | --- | --- | --- | --- |
| 0.24 | -0.03 | 0.04 | 0.02 | -0.10 | 0.14 | 0.10 | 0.13 | 0.04 |
| 0.30 | -0.01 | 0.25 | 0.06 | 0.24 | 0.07 | 0.01 | -0.04 | 0.15 |
| 0.24 | -0.06 | 0.15 | 0.05 | 0.11 | 0.20 | -0.02 | 0.34 | 0.20 |
| 0.08 | 0.15 | 0.34 | -0.07 | 0.06 | 0.06 | 0.08 | 0.26 | 0.13 |
| 0.10 | -0.20 | 0.29 | -0.01 | 0.03 | 0.03 | 0.00 | 0.15 | 0.34 |
| -0.02 | -0.24 | 0.07 | 0.00 | -0.03 | 0.03 | 0.09 | 0.24 | -0.27 |
| 0.23 | -0.17 | 0.08 | 0.00 | -0.30 | -0.08 | 0.06 | 0.30 | 0.12 |
| 0.09 | 0.05 | -0.02 | 0.22 | 0.36 | -0.07 | -0.11 | 0.08 | 0.13 |
| 0.26 | -0.04 | 0.06 | 0.24 | -0.03 | 0.20 | 0.03 | 0.00 | 0.12 |
| -0.02 | -0.04 | 0.00 | 0.21 | 0.15 | 0.03 | 0.39 | -0.12 | -0.02 |
| 0.25 | -0.01 | 0.26 | 0.07 | 0.02 | 0.06 | -0.06 | 0.10 | 0.08 |
| 0.23 | 0.05 | 0.05 | -0.35 | 0.17 | 0.08 | 0.12 | 0.09 | 0.15 |
| -0.12 | 0.06 | -0.16 | 0.08 | 0.00 | 0.06 | 0.05 | -0.11 | -0.16 |
| 0.25 | -0.15 | 0.27 | 0.02 | -0.27 | -0.07 | -0.02 | 0.12 | 0.09 |
| 0.11 | -0.15 | 0.27 | 0.11 | 0.14 | 0.03 | 0.06 | 0.10 | 0.28 |
| 0.01 | 0.14 | -0.09 | 0.19 | 0.21 | 0.04 | -0.05 | -0.05 | -0.01 |
| -0.09 | 0.27 | -0.19 | -0.08 | 0.35 | 0.11 | -0.11 | -0.12 | 0.04 |
| -0.07 | 0.45 | -0.07 | -0.06 | 0.33 | -0.08 | -0.02 | -0.13 | 0.08 |
| -0.11 | -0.01 | -0.18 | 0.14 | -0.14 | 0.14 | 0.01 | -0.04 | -0.12 |
| -0.13 | 0.49 | -0.09 | 0.06 | 0.27 | 0.25 | 0.09 | -0.19 | -0.12 |
| -0.14 | 0.01 | 0.10 | 0.04 | 0.08 | 0.00 | 0.06 | -0.05 | 0.09 |
| -0.02 | -0.03 | 0.29 | -0.01 | 0.03 | 0.07 | 0.08 | 0.06 | 0.22 |
| -0.34 | 0.05 | 0.09 | -0.05 | -0.01 | 0.20 | 0.21 | -0.05 | 0.04 |
| -0.01 | -0.06 | 0.27 | -0.12 | -0.06 | 0.10 | 0.10 | 0.24 | -0.02 |
| 0.09 | -0.25 | 0.26 | 0.26 | 0.01 | 0.03 | 0.07 | 0.39 | 0.16 |
| 0.16 | -0.29 | 0.11 | 0.43 | -0.02 | 0.21 | 0.04 | 0.19 | 0.11 |
| -0.08 | -0.02 | -0.01 | 0.04 | -0.08 | 0.22 | -0.07 | 0.02 | -0.09 |
| -0.07 | -0.14 | 0.16 | -0.03 | 0.09 | -0.06 | -0.03 | 0.30 | 0.10 |
| -0.16 | -0.10 | 0.01 | 0.29 | 0.06 | 0.14 | 0.21 | 0.02 | 0.20 |
| -0.04 | 0.11 | 0.14 | 0.11 | 0.28 | 0.30 | 0.31 | 0.02 | 0.24 |
| 1.00 | -0.08 | 0.08 | 0.01 | -0.01 | -0.06 | -0.04 | 0.33 | 0.12 |
| -0.08 | 1.00 | 0.01 | -0.25 | 0.34 | -0.11 | 0.07 | -0.38 | -0.13 |
| 0.08 | 0.01 | 1.00 | -0.06 | 0.04 | 0.17 | 0.04 | 0.10 | 0.26 |
| 0.01 | -0.25 | -0.06 | 1.00 | 0.06 | 0.06 | -0.05 | 0.05 | 0.23 |
| -0.01 | 0.34 | 0.04 | 0.06 | 1.00 | -0.06 | 0.05 | -0.14 | -0.06 |
| -0.06 | -0.11 | 0.17 | 0.06 | -0.06 | 1.00 | 0.21 | -0.07 | 0.20 |
| -0.04 | 0.07 | 0.04 | -0.05 | 0.05 | 0.21 | 1.00 | -0.02 | 0.09 |
| 0.33 | -0.38 | 0.10 | 0.05 | -0.14 | -0.07 | -0.02 | 1.00 | 0.10 |
| 0.12 | -0.13 | 0.26 | 0.23 | -0.06 | 0.20 | 0.09 | 0.10 | 1.00 |

**1.4 Results of the factor analyses within each of the three alleged dimensions applying (Maximum likelihood extraction technique that assume the sample is randomly selected, so the result can be generalized to a larger population; Varimax orthogonal rotation that assume uncorrelated factors)**

**1.4.1 Cognitive dimension: five extracted factors explained 42.08% variance.**

| **Rotated Factor Matrix^a^** | | | | | |
| --- | --- | --- | --- | --- | --- |
| **Item Number** | **Factor** | | | | |
|  | **1** | **2** | **3** | **4** | **5** |
| 1 | -.022 | .459 | .072 | -.072 | .126 |
| 3 | .083 | .248 | .904 | .164 | -.001 |
| 5 | .791 | .163 | .032 | .073 | -.075 |
| 7 | .171 | .548 | .085 | .140 | .001 |
| 9 | -.051 | .240 | .330 | -.105 | .268 |
| 11 | .001 | .400 | .313 | -.059 | .134 |
| 13 | .343 | -.162 | -.255 | .009 | .354 |
| 15 | .709 | .160 | -.055 | .035 | .185 |
| 22 | .332 | -.136 | .109 | .172 | -.030 |
| 25 | .092 | .131 | .300 | .311 | .472 |
| 28 | .142 | -.075 | .005 | .360 | .112 |
| 31 | .003 | .515 | .123 | .147 | -.059 |
| 34 | .004 | .054 | .023 | .053 | .450 |
| 38 | .018 | .380 | .050 | .923 | .019 |
| Extraction Method: Maximum Likelihood.  Rotation Method: Varimax with Kaiser Normalization. | | | | | |
| a. Rotation converged in 7 iterations. | | | | | |

**1.4.2 Reflective dimension: five extracted factors explained 46.76% variance.**

| **Rotated Factor Matrix^a^** | | | | | |
| --- | --- | --- | --- | --- | --- |
| **Item Number** | **Factor** | | | | |
|  | **1** | **2** | **3** | **4** | **5** |
| **6** | -.074 | .098 | .974 | -.169 | -.077 |
| **10** | .198 | .554 | .035 | -.136 | .050 |
| **16** | .583 | -.051 | -.034 | -.214 | .104 |
| **18** | .663 | -.106 | .030 | .306 | .673 |
| **20** | .605 | .061 | .033 | .120 | -.151 |
| **23** | -.015 | .330 | .304 | .144 | -.007 |
| **26** | -.008 | .148 | .025 | -.434 | -.088 |
| **29** | -.014 | .553 | -.007 | -.204 | -.054 |
| **32** | .574 | .108 | -.084 | .737 | -.273 |
| **35** | .348 | .067 | -.049 | .139 | .077 |
| **37** | -.019 | .593 | .095 | .077 | .053 |
| **39** | -.060 | .232 | -.173 | -.049 | .263 |
| Extraction Method: Maximum Likelihood.  Rotation Method: Varimax with Kaiser Normalization. | | | | | |
| a. Rotation converged in 15 iterations. | | | | | |

**1.4.3 Affective dimension: six extracted factors explained 49.69% variance.**

| **Rotated Factor Matrix^a^** | | | | | | |
| --- | --- | --- | --- | --- | --- | --- |
| **Item Number** | **Factor** | | | | | |
|  | **1** | **2** | **3** | **4** | **5** | **6** |
| **2** | -.143 | .186 | .184 | -.034 | .121 | .539 |
| **4** | .176 | -.038 | .953 | .205 | .092 | -.088 |
| **8** | .105 | -.082 | -.161 | .002 | -.056 | .279 |
| **12** | .003 | .082 | .386 | -.173 | -.022 | .038 |
| **14** | .865 | -.051 | .029 | .042 | .008 | .211 |
| **17** | -.596 | .140 | -.020 | -.058 | -.044 | .033 |
| **19** | .008 | .155 | .062 | .050 | -.226 | .030 |
| **21** | -.197 | -.011 | .052 | -.199 | .219 | -.283 |
| **24** | .301 | .056 | .161 | -.130 | .144 | -.144 |
| **27** | .017 | .139 | -.038 | .987 | -.052 | .018 |
| **30** | -.108 | .291 | .007 | .027 | .056 | .085 |
| **33** | .234 | .247 | .191 | .041 | .917 | .066 |
| **36** | .019 | .982 | .085 | .059 | -.084 | -.126 |
| Extraction Method: Maximum Likelihood.  Rotation Method: Varimax with Kaiser Normalization. | | | | | | |
| a. Rotation converged in 11 iterations. | | | | | | |
