# Supplementary material for: Thin-Slice Measurement of Wisdom
Source: Front Psychol. 2017 Aug 15;8:1378. doi: 10.3389/fpsyg.2017.01378 (PMC5559494; doi:10.3389/fpsyg.2017.01378)
Supplement: Supplementary file 2 [file Image_1.pdf]

Supplementary Material

Adaptation of Western Wisdom Paradigms and development of a 2nd person measurement for Chinese

Chao S. Hu *, Michel Ferrari, Qiandong Wang, Earl Woodruff

*** Correspondence:** Chao S. Hu: chao.super.hu@gmail.com

1 Supplementary Figures

1.1 Scatterplot for the correlation of proportional time of surprise with Chinese wisdom rating.

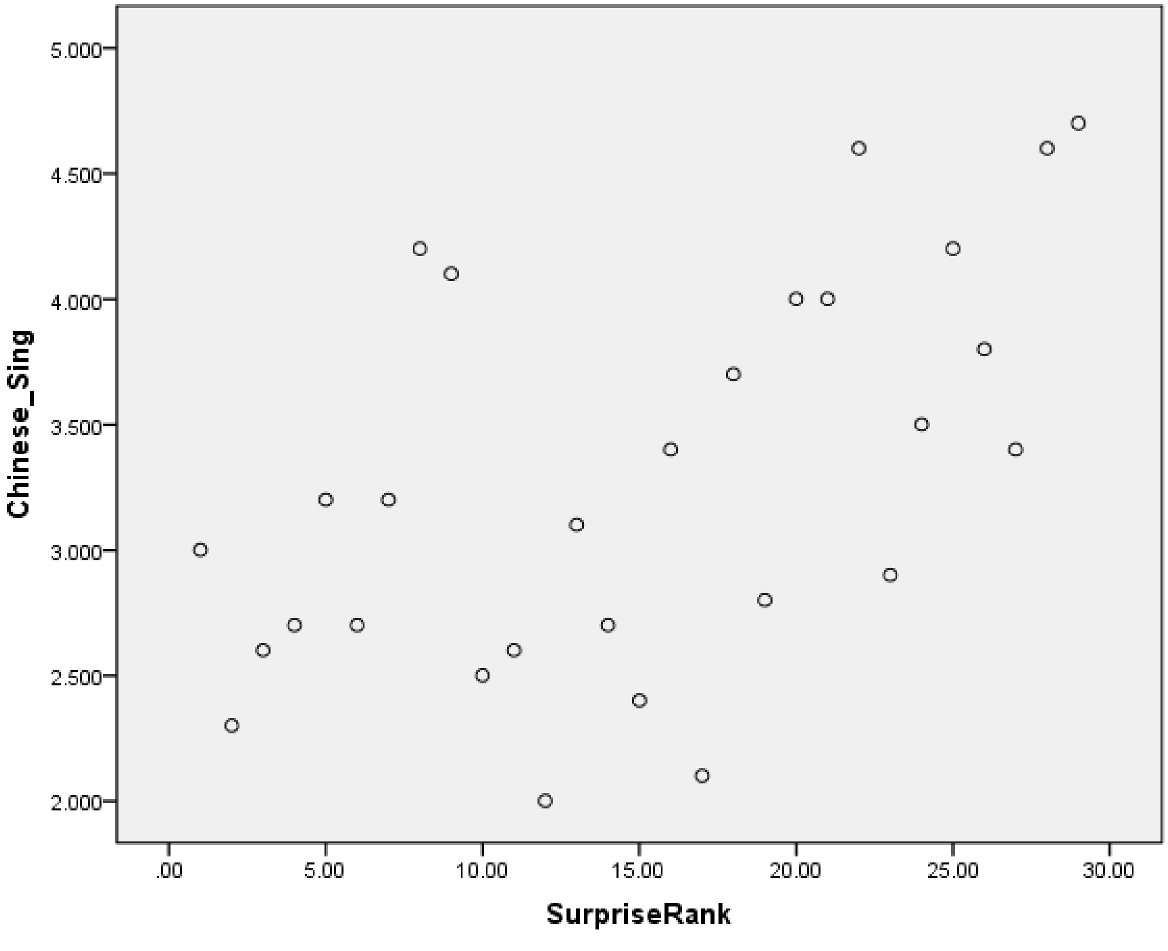

(Note: the rank of the proportional time is used as the X-axis , just as the Spearman correlation analysis was applied, because the distribution of the proportional time data was not normal.)

1.2 Scatterplot for the correlation of proportional time of surprise with Berlin wisdom rating.

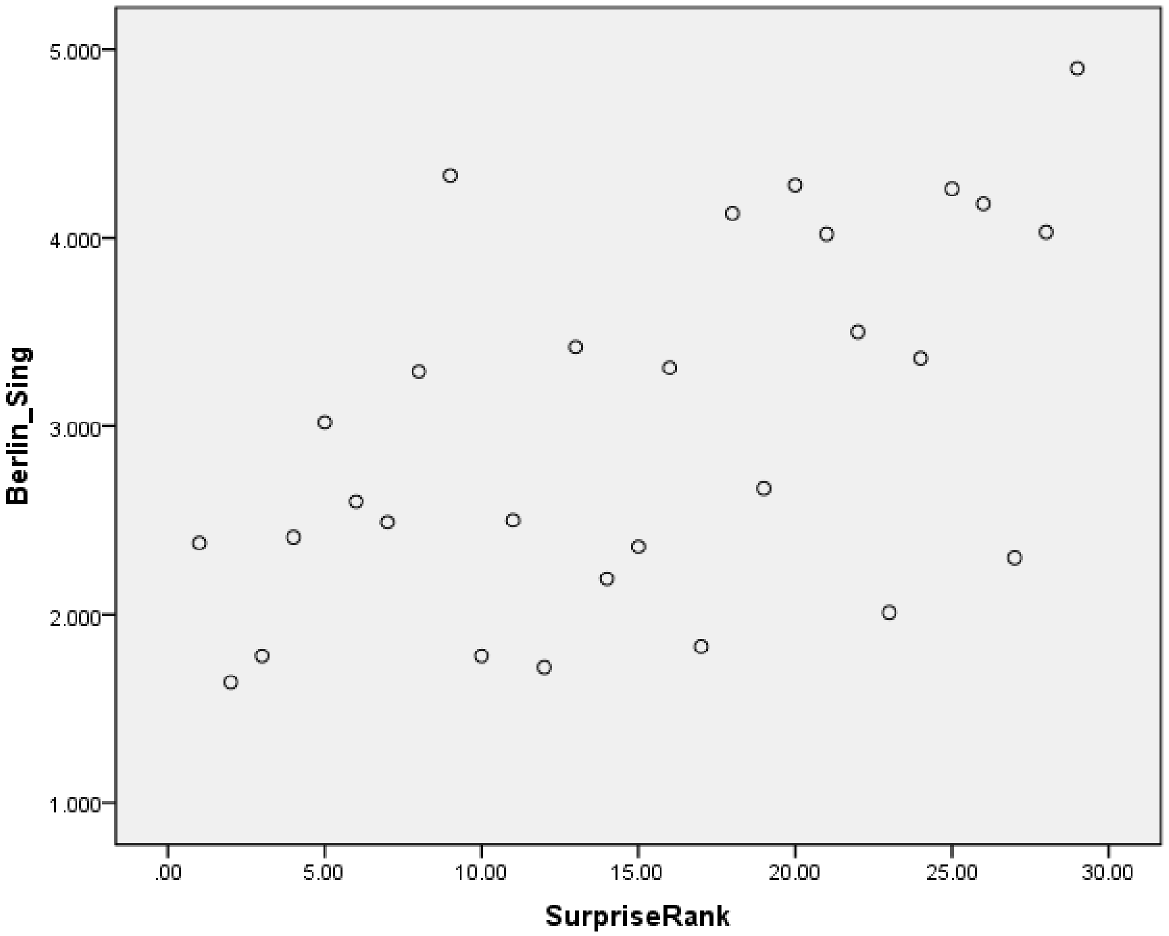

(Note: the rank of the proportional time is used as the X-axis , just as the Spearman correlation analysis was applied, because the distribution of the proportional time data was not normal.)
